# Behavioural patterns only predict concurrent BMI status and not BMI trajectories in a sample of youth in Ontario, Canada

**DOI:** 10.1371/journal.pone.0190405

**Published:** 2018-01-02

**Authors:** Rachel E. Laxer, Martin Cooke, Joel A. Dubin, Ross C. Brownson, Ashok Chaurasia, Scott T. Leatherdale

**Affiliations:** 1 School of Public Health and Health Systems, University of Waterloo, Waterloo ON Canada; 2 Brown School and School of Medicine, Washington University in St. Louis, St. Louis MO, United States of America; Medical University of Vienna, AUSTRIA

## Abstract

**Background:**

Youth are engaging in multiple risky behaviours, increasing their risk of overweight, obesity, and related chronic diseases. The objective of this study was to examine the effect of engaging in unique clusters of unhealthy behaviours on youths’ body mass index (BMI) trajectories.

**Methods:**

This study used a linked-longitudinal sample of Grades 9 and 10 students (13 to 17 years of age) participating in the COMPASS host study. Students reported obesity-related and other risky behaviours at baseline and height and weight (to derive BMI) at baseline (2012/2013) and annually for 2 years post-baseline (2013/14 and 2014/15). Students were grouped into behavioural clusters based on response probabilities. Linear mixed effects models, using BMI as a continuous outcome measure, were used to examine the effect of engaging in clusters of risky behaviours on BMI trajectories.

**Results:**

There were significant differences in BMI of the four behavioural clusters at baseline that remained consistent over time. Higher BMI values were found among youth classified at baseline to be *Typical High School Athletes* (β *=* 0.232 kg/m^2^, [confidence interval (CI): 0.03–0.50]), *Inactive High Screen-User* (β = 0.348 kg/m^2^, CI: 0.11–0.59) and *Moderately Active Substance Users* (β = 0.759 kg/m^2^, CI: 0.36–1.15) compared to students classified as *Health Conscious*. Despite these baseline differences, BMI appeared to increase across all behavioural clusters annually by the same amount (β = 0.6097 kg/m^2^, (CI) = 0.57–0.64).

**Conclusions:**

Although annual increases in BMI did not differ by behavioural clusters, membership in a particular behavioural cluster was associated with baseline BMI, and these differences remained consistent over time. Results indicate that intervening and modifying unhealthy behaviours earlier might have a greater impact than during adolescence. Health promotion strategies targeting the highest risk youth as they enter secondary school might be promising means to prevent or delay the onset of obesity.

## Introduction

There has been a notable increase in the prevalence of both measured and self-reported overweight and obesity over the last 30 years, with approximately 27% of Canadian children and youth now classified as overweight or obese [1]. Obesity in adolescence is associated with an increased risk of adult obesity and other chronic diseases, including cardiovascular disease, diabetes, and hypertension [2]. Overweight youth also tend to be at risk for psychosocial problems, to complete fewer years of higher education, and subsequently to live in households with lower average incomes [2, 3]. There is a need to better understand the causes and correlates of overweight and obesity in adolescence.

The positive energy balance contributing to overweight and obesity through low levels of physical activity and poor dietary behaviours, is often the focus in obesity research [4]. However, total fat and energy intake have remained relatively constant [5], suggesting that other behaviours might be more influential of adolescent weight status. For example, advances in technology have led to a marked increase in screen time and sedentary behaviour among adolescents, which are often coupled with lower energy expenditure. Other risk behaviours that tend to emerge in adolescence, such as alcohol consumption [6] and cigarette smoking have also been linked to an increase in percent body fat, overweight, and obesity [7]. However, the mechanism by which these behaviours contribute to overweight or obesity is not well understood. And while individually linked to an increased risk of overweight and obesity in youth [8, 9], these behaviours do not occur in isolation, but rather cluster in unique ways [10, 11, 12]. Since most youth are not engaging in optimal behavioural patterns [10, 11, 13] and report engaging in at least one modifiable risk behaviour [12], there is a reinforced need for prevention programming targeting youth.

Evidence from cross-sectional studies has demonstrated an association between risky behavioural clusters and obesity among children and youth [11, 12]. While useful for surveillance of youth risk behaviours, cross-sectional data do not provide the necessary data to quantify trajectories at the individual level [14], nor to examine a temporal relationship between risky behavioural patterns and body mass index (BMI). The existing longitudinal research focused on single behaviours, and suggests that among children, behaviours protective of healthy body weights include physical activity [15, 16, 17], sports participation [18], low sedentary behaviours [15, 16, 19, 20], and a healthier diet [21]. However, little is known about other risky behaviours (smoking, marijuana use, binge drinking), or the combined effect of these behaviours on BMI trajectories.

Prevention and intervention programs, frequently developed to target specific behaviours, might be more effective if comprehensive since behaviours rarely occur in isolation [22]. To best target such prevention programming, it is important to understand optimal behaviour patterns and to place emphasis on the strategies that target more complex behavioural patterns rather than single behaviours. Our objectives were to: (1) examine variation in BMI across distinct combinations of risky behaviours in youth and (2) identify if behavioural cluster membership predicted BMI trajectories in youth. Identifying the behavioural clusters associated with an accelerated BMI trajectory might help researchers better allocate resources and direct efforts to target appropriate modifiable behaviours. Steeper BMI trajectories towards overweight and obesity might suggest the importance of earlier interventions to improve the trajectory of BMI in youth.

## Methods

### Design

The COMPASS Study (COMPASS) is a prospective cohort study designed to collect hierarchical and longitudinal data from a sample of secondary school students and the schools that they attend in Ontario and Alberta, Canada. This manuscript used data collected from the cohort of 5,084 students in 41 Ontario schools that participated in the first three years: years 1 (Y_1_: 2012–2013), 2 (Y_2_: 2013–2014), and 3 (Y_3_: 2014–2015) of the COMPASS Host Study. Data were obtained from 41 purposefully sampled Ontario schools that agreed to use active-information, passive-consent parental permission protocols. Student-level data were collected annually using the COMPASS questionnaire. A full description of the COMPASS study and its methods is available online (www.compass.uwaterloo.ca) and in print [23]. The COMPASS study received ethics approval from the University of Waterloo Human Research Ethics Committee, as well as from review panels of all participating school boards.

### Sample and population

**Fig 1:**
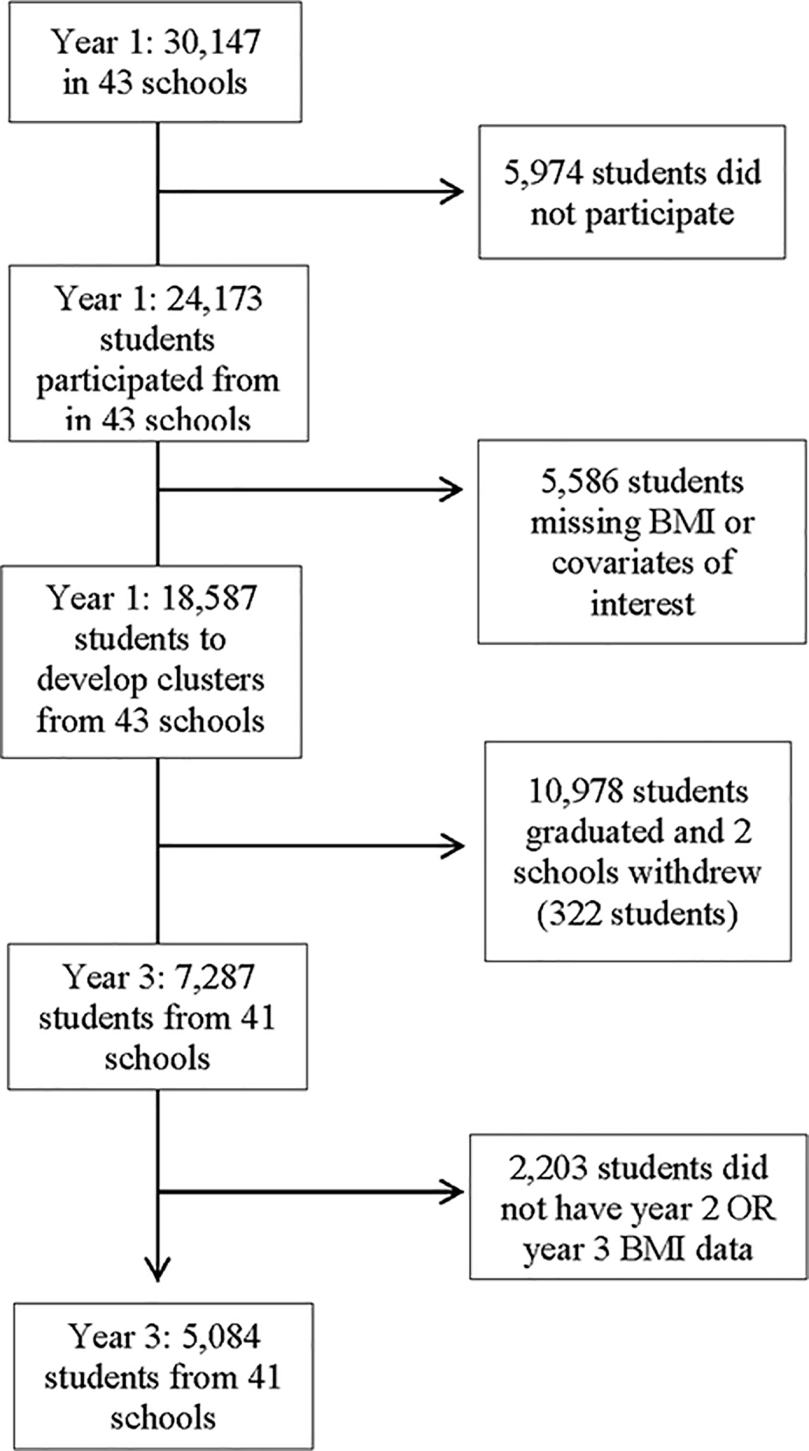
Flowchart of participating students and schools.

In Y_1_, a total of 30,147 students in grades 9 to 12 were enrolled in 43 COMPASS secondary schools. Overall, 80.2% (n = 24,173) of eligible Y_1_ students completed the questionnaire during class time on the day of the scheduled data collection. Non-responses resulted from student absenteeism (19%), parent refusal (0.9%) or student refusal (0.1%). Records missing information on height, weight, or other covariates of targeted interest (gender, race, grade, or spending money) were excluded from the analysis sample. While additional schools were recruited in Y_2_ and Y_3_, only the students that participated in Y_1_ of COMPASS were included in this manuscript. As described elsewhere [24], self-generated identification codes were used to link data sets for the three years and to create the longitudinal data set for analyses. To ensure a sufficient sample size, this study used available-case rather than complete case analysis; complete case analysis involves deleting all cases for which there are any missing values, whereas available case analysis only deletes cases if all but one values are missing [25]. This study therefore included participants that provided behavioural data at Y_1_, and BMI data at Y_1_ and at least one of two years of follow-up (Y_2_ and Y_3_). Between Y_1_ and Y_3_, two schools withdrew from COMPASS, and 10,978 graduated from grade 12 (5,699 in Y_1_ and 5,279 in Y_2_). The final linked longitudinal sample used for this study included 5,084 students from 41 schools in Ontario (see Fig 1).

### Measures

#### Outcome variable–BMI

Students’ self-reported height (in meters) and weight (in kilograms) were used to calculate BMI (in kg/m^2^) using the standard formula. The COMPASS BMI measure was validated in a sample of grade 9 students from Ontario, Canada, and demonstrated substantial 1-week test-retest reliability (Intraclass correlation [ICC] = 0.95) and concurrent validity with measured height and weight (ICC = 0.84) [26]. BMI was classified as a continuous outcome measure, as it can provide more nuanced information than BMI categories when examining changes to BMI over short time periods [27].

#### Predictor variables–risk behaviour clusters

The risk behaviour clusters used for this study are those identified in previous research using Y_1_ COMPASS data [11]. A latent class analysis (LCA) to identify patterns of 15 behavioural indicators associated with overweight and obesity [physical activity (time spent in hard and moderate physical activity, days engaging in strength training, physical activities organized by the school, and participation on a competitive school sports teams), dietary behaviours (breakfast consumption, fast food consumption, snacking behaviour, sugar-sweetened beverage consumption, and fruit and vegetable consumption), sedentary behaviour (time spent watching television, playing video/computer games, and surfing the internet), and substance use behaviours (smoking, marijuana use, and binge drinking)] was conducted on a subsample of youth (n = 18,587) participating in Y_1_ of COMPASS [11]. The LCA identified four unique behavioural clusters: 1) *Traditional School Athletes*; 2) *Inactive High Screen-User*; 3) *Health Conscious*; and, 4) *Moderately Active Substance Users* to which youth were assigned based on highest probability of group membership [11, 28]. Behaviour cluster membership in youth at baseline was used to predict their BMI trajectories. For details on the patterns of behaviours across the four clusters, see Table 1; for more information on the creation of the clusters, see [11].

**Table 1:** Description of the four previously created behavioural clusters.

Latent Class	Description
Health Conscious	- 54.6% met physical activity guidelines. - 59.1% engaged in in strength training at least three times per week - 73.0% participated in varsity and intramural (61.8%) sports. - 81.8% spent less than 2 hours per day surfing the internet per day - 8.5% spent less than 2 hours playing video games per day - 23.6% spent less than 2 hours watching television per day - 51.3% ate at least 5 servings of fruits or vegetables per day - 79.5% ate breakfast daily - 71.6% did not eat at fast food restaurants - 93.6% did not eat snacks from corner stores or drink sugar sweetened beverages - <3.0% smoked or used marijuana - 88.7% did not binge drink
Typical High School Athletes	- 63.6% met physical activity recommendations - 50.5% engaged in strength training - 99.1% belonged to a varsity team - 87.4% played intramural sports - 46.9% surfed the internet more than two hours per day - 59.2% watched television for more than two hours per day - 70.4% did not consume five servings of fruits and vegetables daily - 52.3% did not consume breakfast daily - 81.5% ate at fast food restaurants once or more per week - 45.9% consumed sugar-sweetened beverages at least three times per week. - 10% used marijuana - 25.7% were considered binge drinkers
Inactive High Screen-Users	- 37% met physical activity recommendations - 26.1% participated in strength training at least three times per week - 7.6% participated in varsity sports - 11.2% participated in intramural sports - 58.0% spent more than two hours per day surfing the internet and watching television. - 85% did not consume at least five servings of fruits and vegetables daily - 57% skipped breakfast - 67.7% consumed fast food more than once per week
Moderately Active Substance Users	- 57% spent two or more hours per day surfing the internet and watching television - 74.9% were not consuming breakfast - 76.9% did not consume five or more servings of fruits and vegetables per day - 85% consumed fast food once or more times per wee - 69.8% were smokers - 82.6% were marijuana users - 79.4% were binge drinkers

#### Covariates

Sociodemographic characteristics were measured in youth at Y_1_. Covariates considered in the analyses were found previously to be associated with BMI and health behaviours in youth, and included students’ self-reported gender (male, female), grade (9, 10, 11, 12), race (White, Aboriginal [First Nations, Métis, Inuit], other), and self-reported average weekly spending money ($0, $1-$20, $21-$100, more than $100, “I don’t know”) [29].

### Statistical analysis

All analyses were performed using the statistical package SAS 9.4 (SAS Institute Inc., Cary, NC, USA). Descriptive statistics were calculated for the total sample.

We used PROC MIXED to fit 3-level linear mixed effects models (level 1 –school; level 2 –students within schools; and 3 –repeated BMI measures over time within students) [30]. Using three years of data, the models tested the effects of engaging in risky behaviours at baseline (behaviour cluster membership) on youths’ BMI trajectories. An initial null model was executed to examine variability in the BMI outcome that can be attributed to the clustered nature of the data and to identify if school (as a cluster variable) was necessary to include in the models. Although small, variability across the schools was significant and was considered in all subsequent models. We considered three models of behavioural cluster membership on BMI trajectories, considering BMI as a continuous outcome measure: controlling for year (Model 1), controlling for year, gender, grade, race, and weekly spending money (Model 2), and lastly, Model 2 control variables with test for interaction between time and behavioural cluster (Model 3). All models controlled for year in the analyses. We included two random effects: (1) school and (2) students nested within schools. The remaining effects were fixed (cluster group, gender, grade, ethnicity, money).

## Results

At Y_1_, 17.7% of youth were overweight and 7.9% were obese. As identified in a previous paper (14), the youth that belonged to less healthy behavioural clusters compared to the *Health Conscious* cluster were considered to be at an increased risk of overweight and obesity. Details on the identification of the four behavioural clusters are described elsewhere [11].

### Description of the study sample

Participant characteristics for the total linked sample (n = 5,084) and by behavioural clusters can be found in Table 2. Approximately half of the students were female (52.1%), a large majority were White (75.8%), and most were in Grades 9 (46.5%) or 10 (50.9%). The behavioural cluster most strongly represented in this sample was the *Inactive High Screen-Users* (44.9%), while the *Moderately Active Substance Users* was the least represented (7.8%). The mean BMI of the total sample at baseline was 21.3 kg/m^2^, with the lowest BMI found among the *Health Conscious* youth (20.9 kg/m^2^) and the highest among the *Moderately Active Substance Users* (22.1 kg/m^2^).

**Table 2:** Baseline characteristics of the linked-longitudinal sample of youth participating in Y_1_ to Y_3_ of the COMPASS study in Ontario, Canada (2012–2015).

Variable	Total (n = 5084)	Typical High School Athlete (n = 1419, 27.9%)	Inactive High Screen-Users (n = 2285, 44.9%)	Health Conscious (n = 1008, 19.8%)	Moderately Active Substance Users (n = 372, 7.3%)
Gender, n(%)*					
Males	2438 (48.0)	582 (22.0)	1341 (50.7)	570 (21.5)	153 (5.8)
Females	2646 (52.1)	837 (34.3)	944 (38.7)	438 (18.0)	219 (9.0)
**Race, n(%)**					
White	3854 (75.8)	1095 (28.4)	1678 (43.5)	807 (20.9)	274 (7.1)
Aboriginal	170 (3.3)	23 (22.1)	43 (41.4)	16 (15.3)	22 (21.2)
Other	1060 (20.9)	301 (26.7)	564 (50.1)	185 (16.4)	76 (6.8)
**Grade, n(%)**					
9	2363 (46.5)	653 (27.6)	1084 (45.9)	514 (21.8)	112 (4.7)
10	2588 (50.9)	730 (28.2)	1139 (44.0)	476 (18.4)	243 (9.4)
11	133 (2.6)	36 (27.3)	62 (46.1)	18 (14.1)	17 (12.5
Age (m, d)*	14.7 (0.67)	14.7 (0.67)	14.66 (0.68)	14.68 (0.69)	14.94 (0.68)
**Spending money, n(%)**					
None	939 (18.5)	203 (21.6)	472 (50.3)	232 (24.7)	32 (3.4)
$1 to $20	1917 (37.7)	526 (27.4)	935 (48.8)	344 (17.9)	112 (5.8)
$21 to $100	1246 (24.5)	389 (31.2)	511 (41.0)	220 (17.7)	126 (10.1)
More than $100	326 (6.4)	99 (30.4)	100 (30.7)	61 (18.7)	66 (20.3)
I do not know	656 (12.9)	202 (30.8)	267 (40.7)	151 (23.0)	36 (5.5)
**BMI (m, d)**	21.3 (3.36)	21.3 (3.18)	21.3 (3.54)	20.9 (3.07)	22.1 (3.46)

* (m,d), mean (standard deviation); n(%), number (percentage)

**Fig 2:**
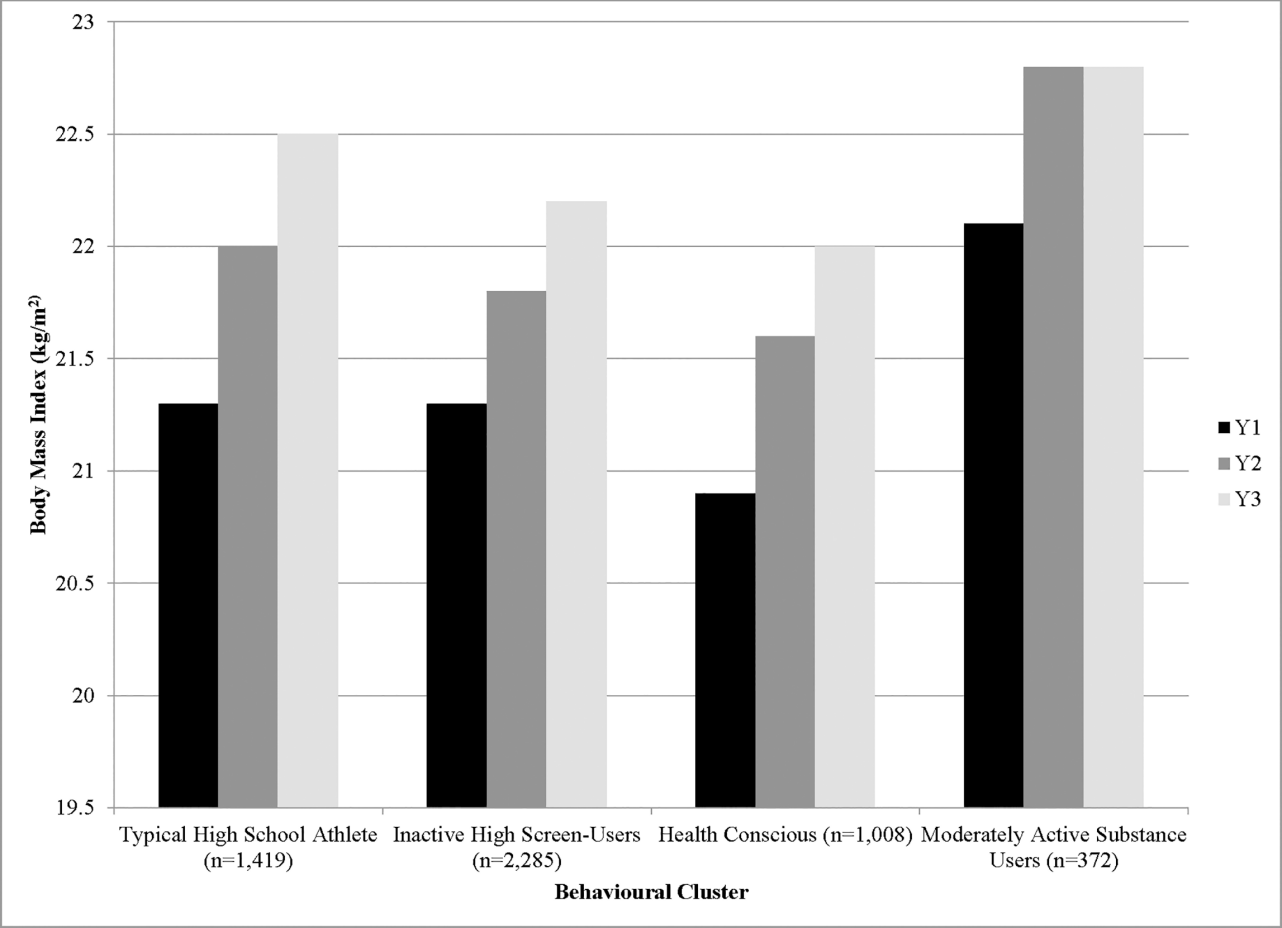
Average annual changes in BMI from the sample participating in Y_1_ to Y_3_ of the COMPASS study in Ontario, Canada.

Mean BMI increased from 21.3 kg/m^2^ in Y_1_ to 22.3 kg/m^2^ in Y_3_. Males’ self-reported BMIs (21.7–23.0 kg/m^2^) were consistently higher than females’ (20.9–21.7 kg/m^2^). Fig 2 shows that there was an annual increase in average BMI (0.4 to 1.2 kg/m^2^) from Y_1_ to Y_3_ across all behavioural clusters, with an apparent plateau of BMI among the *Moderately Active Substance Users* from Y_2_ to Y_3_.

### Mixed effects regression model results

**Fig 3:**
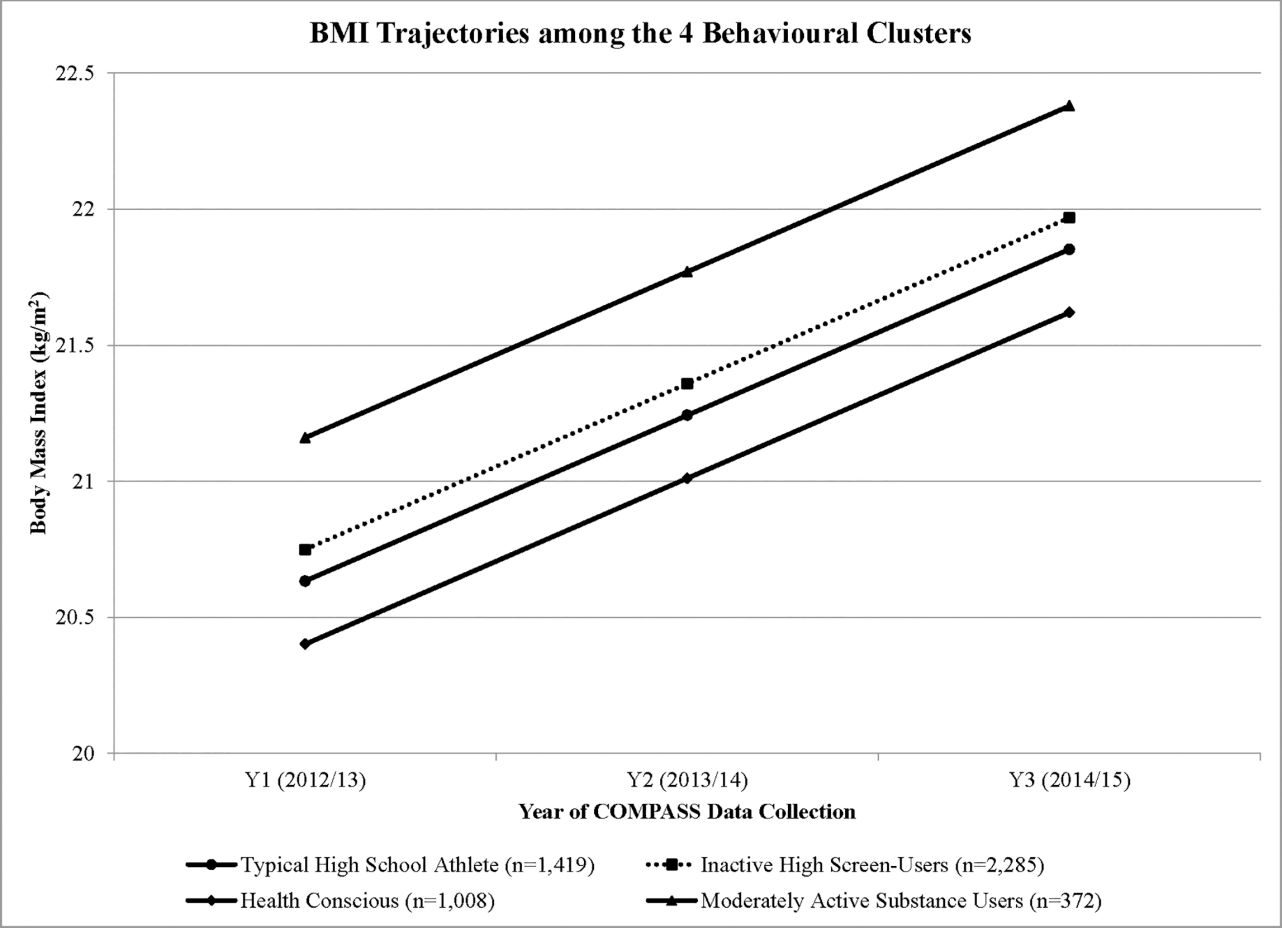
Model-based predicted BMI trajectories of the 4 behavioural clusters from youth participating in Y_1_ to Y_3_ of the COMPASS study in Ontario, Canada.

Regression coefficients for all models are found in Table 3. The empty model used to determine the intraclass correlation identified that there was a cluster effect at the school-level that needed to be considered in all subsequent models (ICC = 2%). Results from Model 1, which only controlled for time and school, suggested that all three behavioural clusters were significantly different from the *Health Conscious* cluster, with differences ranging from 0.344 kg/m^2^ (*Inactive High Screen-Users*) to 1.041 kg/m^2^ (*Moderately Active Substance Users)*. In model 2, controlling for sociodemographic factors, BMIs at baseline for youth belonging to the *Traditional High School Athletes* cluster (20.63 kg/m^2^, confidence interval (CI) 0.03–0.50) was higher than youth in the *Health Conscious* cluster (β = +0.232 kg/m^2^). Similarly, at baseline, BMIs of youth classified as *Inactive High Screen-Users* (20.57 kg/m^2^, 0.11–0.59) and *Moderately Active Substance Users* (22.16 kg/m^2^, 0.36–1.15) were higher compared to youth classified as *Health Conscious* (β = +0.348; β = +0.759 kg/m^2^, respectively). Model 3 results, which incorporated a time* cluster interaction, were not significant, suggesting that the BMI trajectories of youth belonging to different behavioural clusters were not significantly different. Accordingly, results indicate that if all youth continue to engage in the same patterns of health behaviours as identified at, we would predict an increase in BMI of 0.61 kg/m^2^ annually (β = 0.610 kg/m^2^, 0.57–0.64). These projected BMI increases for each cluster are depicted in Fig 3.

**Table 3:** Regression coefficients for the relationship between risky behavioural clusters at baseline and BMI over time among youth participating in Y_1_ to Y_3_ of the COMPASS study in Ontario, Canada (2012–2015).

	Model 1			Model 2		
	B	SE(B)	95% CI	Β	SE(B)	95% CI
Intercept	21.053	0.127	20.8–21.3**	20.401	0.170	20.1–20.7
Time	0.609	0.017	0.58–0.64**	0.610	0.0170	0.57–0.64**
***Cluster group***						
Traditional High School Athletes	0.410	0.136	0.12–0.68*	0.232	0.135	-0.03–0.50
Inactive High Screen-Users	0.344	0.125	0.10–0.59*	0.348	0.124	0.11–0.59*
Health Conscious (ref)	—	—	—	—	—	—
Moderately Active Substance Users	1.041	0.201	0.65–1.44**	0.759	0.202	0.36–1.15*
***Gender***						
Male	—	—	—	1.012	0.093	0.83–1.19**
Female (ref)	—	—	—	—	—	—
***Grade***						
9 (ref)	—	—	—	—	—	—
10	—	—	—	0.401	0.094	0.22–0.58**
11	—	—	—	0.815	0.298	0.23–1.40
***Race***						
White (ref)	—	—	—	—	—	—
Aboriginal	—	—	—	0.558	0.336	-0.10–1.22
Other	—	—	—	-0.023	0.1158	-0.25–0.20
***Weekly Spending money***						
$0 (ref)	—	—	—	—	—	—
$1 to $20	—	—	—	0.012	0.130	-0.24–0.27
$21 to $100	—	—	—	0.014	0.143	-0.27–0.29
More than $100	—	—	—	0.116	0.214	-0.46–0.19
I do not know				-0.134	0.166	-0.30–0.54

B reported for unstandardized coefficients. SE: standard error. 95% CI: 95% Confidence intervals; “ref” = referent category for analyses

** p < .0001

* p < .05

## Discussion

This study investigated the impact of engaging in four distinct clusters of behaviours (*Typical High School Athlete*, *Inactive High Screen-Users*, *Health Conscious*, *and Moderately Active Substance Users*) on the BMI and BMI trajectories of a large sample of youth from Ontario, Canada. Consistent with previous research [31], there were significant differences in the average BMI at baseline across the four behavioural clusters, suggesting that BMI was associated with concurrent weight status. Despite baseline differences, the BMI trajectories for youth in all clusters was 0.610 kg/m^2^ annually, irrespective of their behavioural cluster, thus suggesting that engaging in risky behaviours might only predict BMI at baseline and not differences in trajectories over time. Results did not extend previous research on the correlates contributing to accelerated BMI trajectories, but did confirm that males and older youth have higher BMI trajectories than their counterparts. Efforts are required to improve health behaviours to slow BMI trajectories in all youth belonging to all clusters, considering the heterogeneity of BMI at baseline and noting that some subpopulations might develop overweight or obesity earlier than others based on their baseline BMI [14].

The limited longitudinal research to date on health behaviours and BMI trajectories has been mixed. Some have found that unhealthy weight-related behaviours, including higher caloric consumption, lower physical activity, and higher screen time, are associated with larger increases in BMI over time [16, 32, 33], and that modifying these risk behaviours can improve BMI [17, 34]. Others found that physical activity in adolescence did not predict obesity five years later, but that decreases in screen time during in adolescence were associated with lower rates of obesity [35]. This was opposite to research by Chinapaw et al., which found that sedentary time in youth was not related to BMI [36]. However, Chinapaw only focused on television viewing behaviours, which may have underestimated youths’ true screen-based behaviours (i.e., video games, cell phone use, internet surfing) [37]. In terms of dietary behaviours, some have identified that energy intake was inversely related to fat mass, opposite to what would be expected based on the theory of energy balance [38, 39]. The general consensus from this previous research is a need to investigate a potential combined effect of engaging in multiple behaviours on BMI, rather than focusing on individual behaviours.

The results of this study suggest that all risk behaviour clusters require attention. The projected BMI trajectories depicted in Fig 3 starting with baseline BMI for all behaviorual clusters demonstrates the increase in BMI that each cluster would experience over time, with no behavioural interventions. These estimates might be conservative, as we did not consider the likely increase of risk factor prevalence and adoption of other risky behaviours as youth leave high school and transition to college, university, or the workforce [40]. Interestingly, the *Moderately Active Substance Users’* BMIs, as seen in Fig 2, appeared to plateau after Y_2_. This study used an available case analysis, including students with at least 2 years of BMI data. Many of the missing students in Y_3_ belonged to the *Moderately Active Substance Users* cluster. Such characteristics (smoking, marijuana use, binge drinking) are typically embodied by students that tend to skip school and may not have been present on the day of the survey [41]. As such, the leveling off of BMI in this cluster group may be explained by the missing data.

This study focused on the effect of baseline behavioural cluster membership on BMI trajectories. The use of behaviours at baseline assumed that behaviours either remain consistent over time, or that the effects of behaviours on BMI might be lagged. Using latent transition models, a method that allows researchers to examine movement between subgroups and how membership in subgroups might change over time [42], researchers demonstrated that health behaviours and the ways in which behaviours cluster tend to remain consistent over time in youth [43], and that BMI trajectories established during early-adolescence (aged 8–14 years) remain stable over time among children who are heavier. With this sample, measuring health behaviour clusters as time-variant between baseline and follow-up might better predict BMI trajectories [44]. Considering other factors as potential moderators, such as body image concerns, weight stigma, or baseline weight status [45, 46], might also help to explain the effects of self-reported behaviours on BMI trajectories. For example, in a study of adolescent weight status and health-related quality of life, adolescent weight perception significantly moderated the relationship between overweight, obesity, and health-related quality of life. Youth that misperceived their weight status to be healthy when they were classified as overweight or obese reported a higher health related quality of life than those youth with weight perceptions concordant with their actual weight status [47].

Since there are few known treatments for reducing or maintaining BMI, a better understanding of the behavioural clusters that most strongly predict BMI at baseline or BMI trajectories might help steer such prevention strategies. This study did not provide evidence to suggest which behavioural clusters are associated with an accelerated BMI trajectory, since time was the only significant predictor of BMI trajectories among all behavioural clusters. Thus, based on these results, it seems that interventions may be warranted for all groups, targeting the risky behaviours that might be present in all clusters, or the behavioural clusters most strongly associated with concurrent weight status. Given limited resources, public health practitioners and researchers should still be purposeful in their prevention planning, by targeting obesity through a comprehensive and multi-sectoral response, one that is capable of targeting co-occurring risky behaviours. These are especially important behaviours to target in childhood or adolescence, because once behavioural patterns are established, they are difficult to modify [48].

Most youth in Canada are not meeting guidelines for healthy diets and healthy physical activity [49], and a large proportion are engaging in other risky behaviours, including marijuana use, smoking, and binge drinking [13]. All youth in this study from all behavioural clusters would require attention, but each cluster would benefit from a different type of intervention. Targeting the *Moderately Active Substance Users* is a novel approach; while substance use is generally not a focus of obesity prevention, it does tend to co-occur with other risky behaviours, and there is rarely focus on substance use for obesity prevention. In fact, those that binge drink consume an excess of calories [6], supporting the idea that alcohol may be partially responsible for driving the increase in BMI [6, 50]. The excess calories may be acquired through the actual alcohol itself, or through the consumption of other unhealthy foods, which often occurs with binge drinking [50]. Researchers might steer away from only focusing on physical activity and dietary behaviour interventions, and instead look towards reducing substance use (i.e., binge drinking) as a potentially effective and novel approach to addressing youth obesity [51]. The notion that targeting efforts towards substance users might be an effective way to prevent or reduce obesity is relevant and timely, given the potentially easy access that youth have to substances–Canada is in the process of legalizing marijuana and Ontario recently began to sell beer in grocery stores [52]. Such a targeted approach would require evaluation through ongoing data collection and evaluation systems, such as COMPASS, to evaluate if such policies and natural experiments have unintended consequences on the BMI trajectories of youth.

Although the magnitude of the estimated effects might appear small, they resemble those of earlier longitudinal studies [16, 17], and can lead to substantial increases in BMI and fat mass if sustained over time. Results emphasize the need to promote healthy behaviours among youth from a younger age, while behaviours are beginning to develop and are likely to sustain. Researchers might begin with targeting healthy eating first by promoting the importance of and access to increased fruit and vegetable consumption and an increase in breakfast consumption. [17, 21, 53]. However, the results of this study must be interpreted with caution, since the mean BMI was similar across all four groups, suggesting they may not be sufficiently different to draw true comparisons. It must also be noted that the causal relationship in this study may not be entirely explained by the behavioural clusters influencing BMI trajectories, rather that youths’ weight status might be the cause of certain types of behavioural patterns. For example, youth with overweight or obesity might be less active for fear of stigmatization from their peers, choosing rather to spend their time in screen-based activities [54].

### Strengths and limitations

This study has several strengths. Its main strength is its longitudinal design, which could have contributed to the causality assumption that risky obesity-related health behaviours are associated with BMI trajectories in a large sample of adolescents. Second, and perhaps also a limitation, was the use of available cases instead of completed cases [25], thus providing a less biased estimate of the effect sizes. The two measures might be equally biased if the missing data were unrelated to the questions of interest, which can never be guaranteed in research on BMI [26]. Another strength of this study was the use of BMI as a continuous measure, thus avoiding potential misclassification of obesity that may occur if there was systematic bias in the self-reported height and weight. Further, BMI as a continuous measure is more meaningful in longitudinal research [19], as it considers the entire range of adiposity [16], and can provide a clearer indication of change [19], since it avoids cut-points that are used to define overweight and obesity. Finally, the analytical approach used for this study accounted for the correlation between repeated measurements on the same subjects [55], examined individual and area-level variables in one model to account for clustering of observations, and examining variation between individuals and groups, simultaneously [55].

Some limitations must be considered in light of the study’s strengths. First, all data in COMPASS are self-reported, which might be subject to social desirability or recall biases. Subjects were assured anonymity when completing the survey, and the majority of measures were found to have acceptable reliability and validity. This is most noteworthy for our outcome measure of BMI, which although crude, demonstrated strong reliability and validity, and is the most feasible method for large cohort studies of youth. And since the purpose of this study was to identify how BMI changes over time, it is expected that youth will misreport consistently over time [26]. However, the misreporting is most often an overestimation of height and an underestimation of weight, leading to a “flat slope syndrome,” which would underestimate the proportion of youth on a trajectory toward overweight or obesity [26, 56]. Second, the health behaviour clusters were identified in a previous study based on the behavioural response patterns of students in COMPASS Y_1_ using latent class analysis. And since behavioural clusters through latent class analysis are determined based on one’s highest probability of cluster membership, it is possible that there was overlap and students were assigned to the wrong category. Similarly, the use of the latent classes from a previous study assumed that behaviours remained static and that youth did not transition in or out of other behavioural clusters. Future analyses might consider using a latent trajectory analysis to examine the behavioural clusters over time, concurrently with changes to BMI. Third, although BMI was the most feasible outcome measure for this study, it may not have been the most practical. As such, interpreting the BMI of the different behavioural clusters must be done with caution; youth belonging to the *Inactive High Screen-Users* cluster are more likely to have a higher fat mass, while the *Typical High School Athletes* are more likely to have a higher muscle mass, and both possibly have the same BMI [57]. Thus, reducing BMI in the two groups would have different consequences–among the *Inactive High Screen-Users*, it would mean a reduction in fat mass, while in the *Typical High School Athletes*, it would suggest a reduction in muscle mass, a counterproductive message when the intention is promote healthy behaviours (i.e., physical activity).

## Conclusions and implications

Having insight into how health behaviours cluster together in unique ways to influence BMI or change BMI trajectories might assist in the development of more targeted interventions, especially since the health behaviours comprising the behavioural clusters are modifiable and can be the subject of health promotion programs. However, since behavioural cluster membership was not associated with BMI trajectories, the message to send to program planners is not clear. BMI values across the four clusters did differ significantly at baseline and were predicted to remain different over time. Researchers should consider addressing these modifiable behaviours at an earlier age, before they begin to emerge and cluster together. Modifying behaviours once they are established is difficult; therefore, establishing healthy lifestyle behaviours and behavioural patterns earlier in life should be an important public health priority.

## References

[1] Rodd C, Sharma AK. Recent trends in the prevalence of overweight and obesity among Canadian children. Canadian Medical Association Journal. 2016 9 20;188(13):E313–20. doi: 10.1503/cmaj.150854 27160875 10.1503/cmaj.150854 PMC5026530

[2] Reilly JJ, Methven E, McDowell ZC, Hacking B, Alexander D, Stewart L et al Health consequences of obesity. Arch Dis Child. 2003 9 1; 88(9):748–52. doi: 10.1136/adc.88.9.748 12937090 10.1136/adc.88.9.748 PMC1719633

[3] Gortmaker S, Must A, Perrin J, Sobol A, Dietz W. Social and economic consequences of overweight in adolescence and young adulthood. New England Journal of Medicine. 1993;329(14):1008–1012. doi: 10.1056/NEJM199309303291406 8366901 10.1056/NEJM199309303291406

[4] Dietz WH, Gortmaker SL. Preventing obesity in children and adolescents. Annu Rev Public Health. 2001 5;22(1):337–53. 11274525 10.1146/annurev.publhealth.22.1.337

[5] Troiano R. P., Briefel R. R., Carroll M. D., & Bialostosky K. (2000). Energy and fat intakes of children and adolescents in the United States: data from the National Health and Nutrition Examination Surveys. *The American journal of clinical nutrition*, 72(5), 1343s–1353s. 11063476 10.1093/ajcn/72.5.1343s

[6] Battista K, Leatherdale ST. Estimating how extra calories from alcohol consumption are likely an overlooked contributor to youth obesity. Health Promot Chronic Dis Prev Can. 2017 6;37(6):194 doi: 10.24095/hpcdp.37.6.03 28614047 10.24095/hpcdp.37.6.03 PMC5650014

[7] Pasch KE, Velazquez CE, Cance JD, Moe SG, Lytle LA. Youth substance use and body composition: does risk in one area predict risk in the other? Journal of Youth and Adolescence. 2012 1 1;41(1):14–26. doi: 10.1007/s10964-011-9706-y 21853355 10.1007/s10964-011-9706-y PMC3617983

[8] Sallis JF, Prochaska JJ, Taylor WC. A review of correlates of physical activity of children and adolescents. Med Sci Sports Exerc. 2000 5 1;32(5):963–75. 10795788 10.1097/00005768-200005000-00014

[9] Nelson MC, Neumark-Stzainer D, Hannan PJ, Sirard JR, Story M. Longitudinal and secular trends in physical activity and sedentary behavior during adolescence. Pediatrics. 2006 12 1;118(6):e1627–34. doi: 10.1542/peds.2006-0926 17142492 10.1542/peds.2006-0926

[10] Leech RM, McNaughton SA, Timperio A. The clustering of diet, physical activity and sedentary behavior in children and adolescents: a review. Int J Behav Nutr Phys Act. 2014 1 22;11(1):4. 24450617 10.1186/1479-5868-11-4 PMC3904164

[11] Laxer RE, Brownson RC, Dubin JA, Cooke M, Chaurasia A, Leatherdale ST. Clustering of risk-related modifiable behaviours and their association with overweight and obesity among a large sample of youth in the COMPASS study. BMC public health. 2017 1 21;17(1):102 doi: 10.1186/s12889-017-4034-0 28109270 10.1186/s12889-017-4034-0 PMC5251243

[12] Carson V, Faulkner G, Sabiston CM, Tremblay MS, Leatherdale ST. Patterns of movement behaviors and their association with overweight and obesity in youth. Int J Public Health. 2015 7 1;60(5):551–9. doi: 10.1007/s00038-015-0685-8 25985847 10.1007/s00038-015-0685-8

[13] Leatherdale ST. An examination of the co-occurrence of modifiable risk factors associated with chronic disease among youth in the COMPASS study. Cancer Causes Control. 2015 4 1;26(4):519–28. doi: 10.1007/s10552-015-0529-0 25673505 10.1007/s10552-015-0529-0

[14] Nonnemaker JM, Morgan‐Lopez AA, Pais JM, Finkelstein EA. Youth BMI trajectories: evidence from the NLSY97. Obesity. 2009 6 1;17(6):1274–80. doi: 10.1038/oby.2009.5 19584884 10.1038/oby.2009.5

[15] Moore LL, Gao D, Bradlee ML, Cupples LA, Sundarajan-Ramamurti A, Proctor MH, et al Does early physical activity predict body fat change throughout childhood? Preventive medicine. 2003 7 31;37(1):10–7. 12799124 10.1016/s0091-7435(03)00048-3

[16] Berkey CS, Rockett HR, Field AE, Gillman MW, Frazier AL, Camargo CA, Colditz GA. Activity, dietary intake, and weight changes in a longitudinal study of preadolescent and adolescent boys and girls. Pediatrics. 2000 4 1;105(4):e56–. 10742377 10.1542/peds.105.4.e56

[17] Elgar FJ, Roberts C, Moore L, Tudor-Smith C. Sedentary behaviour, physical activity and weight problems in adolescents in Wales. Public health. 2005 6 30;119(6):518–24. doi: 10.1016/j.puhe.2004.10.011 15826893 10.1016/j.puhe.2004.10.011

[18] Kemper HC, Post GB, Twisk JW, Van Mechelen W. Lifestyle and obesity in adolescence and young adulthood: results from the Amsterdam Growth and Health Longitudinal Study (AGAHLS). Int J Obes. 1999 4 2;23. 10.1038/sj.ijo.0800881 10368000

[19] Must A, Tybor DJ. Physical activity and sedentary behavior: a review of longitudinal studies of weight and adiposity in youth. Int J Obes. 2005 9 1;29:S84–96. 10.1038/sj.ijo.0803064 16385758

[20] Burke V, Beilin LJ, Simmer K, Oddy WH, Blake KV, Doherty D, et al Predictors of body mass index and associations with cardiovascular risk factors in Australian children: a prospective cohort study. Int J Obes. 2005 1 1;29(1):15–23. 10.1038/sj.ijo.0802750 15314630

[21] Haerens L, Vereecken C, Maes L, De Bourdeaudhuij I. Relationship of physical activity and dietary habits with body mass index in the transition from childhood to adolescence: a 4-year longitudinal study. Public Health Nutrition. 2010 10;13(10A):1722–8. doi: 10.1017/S1368980010002284 20883572 10.1017/S1368980010002284

[22] Michie S, van Stralen MM, West R. The behaviour change wheel: a new method for characterising and designing behaviour change interventions. Implement Sci. 2011 4 23;6(1):42. 21513547 10.1186/1748-5908-6-42 PMC3096582

[23] Leatherdale ST, Brown KS, Carson V, Childs RA, Dubin JA, Elliott SJ, et al The COMPASS study: a longitudinal hierarchical research platform for evaluating natural experiments related to changes in school-level programs, policies and built environment resources. BMC Public Health. 2014 4 8;14(1):331. 24712314 10.1186/1471-2458-14-331 PMC4234205

[24] Qian W, Battista K, Bredin C, Stephen Brown K, Leatherdale ST. Assessing longitudinal data linkage results in the COMPASS study COMPASS Technical Report Series. Waterloo: University of Waterloo; 2015.

[25] Baraldi AN, Enders CK. An introduction to modern missing data analyses. Journal of school psychology. 2010 2 28;48(1):5–37. doi: 10.1016/j.jsp.2009.10.001 20006986 10.1016/j.jsp.2009.10.001

[26] Leatherdale ST, Laxer RE. Reliability and validity of the weight status and dietary intake measures in the COMPASS questionnaire: are the self-reported measures of body mass index (BMI) and Canada’s food guide servings robust? Int J Behav Nutr Phys Act. 2013 4 5;10(1):42. 23561578 10.1186/1479-5868-10-42 PMC3663698

[27] Ruel E, Reither EN, Robert SA, Lantz PM. Neighborhood effects on BMI trends: Examining BMI trajectories for Black and White women. Health & Place. 2010 3 31;16(2):191–8. 19879795 10.1016/j.healthplace.2009.09.009 PMC5619238

[28] Lanza ST, Collins LM, Lemmon DR, Schafer JL. PROC LCA: A SAS procedure for latent class analysis. Structural Equation Modeling. 2007 10 23;14(4):671–94. 19953201 10.1080/10705510701575602 PMC2785099

[29] Delva J, Johnston LD, O’Malley PM. The epidemiology of overweight and related lifestyle behaviors: racial/ethnic and socioeconomic status differences among American youth. Am J Prev Med. 2007 10 31;33(4):S178–86. 17884566 10.1016/j.amepre.2007.07.008

[30] Laird NM, Ware JH. Random-effects models for longitudinal data. Biometrics. 1982 12 1:963–74. 7168798

[31] Singh AS, Mulder C, Twisk JW, Van Mechelen W, Chinapaw MJ. Tracking of childhood overweight into adulthood: a systematic review of the literature. Obesity Rev. 2008 9 1;9(5):474–88. 10.1111/j.1467-789X.2008.00475.x 18331423

[32] Berkey CS, Rockett HR, Gillman MW, Field AE, Colditz GA. Longitudinal study of skipping breakfast and weight change in adolescents. Int J Obes. 2003 10 1;27(10):1258–66. 10.1038/sj.ijo.0802402 14513075

[33] Mitchell JA, Pate RR, Beets MW, Nader PR. Time spent in sedentary behavior and changes in childhood BMI: a longitudinal study from ages 9 to 15 years. Int J Obes. 2013 1 1;37(1):54–60. 10.1038/ijo.2012.41 22430304

[34] Gordon‐Larsen P, Adair LS, Popkin BM. Ethnic differences in physical activity and inactivity patterns and overweight status. Obesity. 2002 3 1;10(3):141–9. 10.1038/oby.2002.23 11886936

[35] Boone JE, Gordon-Larsen P, Adair LS, Popkin BM. Screen time and physical activity during adolescence: longitudinal effects on obesity in young adulthood. Int J Behav Nutr Phys Act. 2007 6 8;4(1):26. 17559668 10.1186/1479-5868-4-26 PMC1906831

[36] Chinapaw MJ, Proper KI, Brug J, Van Mechelen W, Singh AS. Relationship between young peoples' sedentary behaviour and biomedical health indicators: a systematic review of prospective studies. Obesity Rev. 2011 7 1;12(7). 10.1111/j.1467-789X.2011.00865.x 21438990

[37] Leatherdale ST. Factors associated with communication-based sedentary behaviors among youth: are talking on the phone, texting, and instant messaging new sedentary behaviors to be concerned about? J Adolesc Health. 2010 9 30;47(3):315–8. doi: 10.1016/j.jadohealth.2010.02.012 20708574 10.1016/j.jadohealth.2010.02.012

[38] Fulton JE, Dai S, Steffen LM, Grunbaum JA, Shah SM, Labarthe DR. Physical activity, energy intake, sedentary behavior, and adiposity in youth. Am J Prev Med. 2009 7 31;37(1):S40–9. 19524155 10.1016/j.amepre.2009.04.010 PMC5821119

[39] Patrick K, Norman GJ, Calfas KJ, Sallis JF, Zabinski MF, Rupp J, et al Diet, physical activity, and sedentary behaviors as risk factors for overweight in adolescence. JAMA Pediatr. 2004 4 1;158(4):385–90. 10.1001/archpedi.158.4.385 15066880

[40] White HR, McMorris BJ, Catalano RF, Fleming CB, Haggerty KP, Abbott RD. Increases in alcohol and marijuana use during the transition out of high school into emerging adulthood: The effects of leaving home, going to college, and high school protective factors. J Stud Alcohol Drugs. 2006 11;67(6):810–22. 10.15288/jsa.2006.67.810 PMC2314672 17060997

[41] Henry KL. Who’s skipping school: Characteristics of truants in 8th and 10th grade. J Sch Health. 2007 1 1;77(1):29–35. doi: 10.1111/j.1746-1561.2007.00159.x 17212757 10.1111/j.1746-1561.2007.00159.x

[42] Collins LM, Lanza ST. Latent class and latent transition analysis: With applications in the social, behavioral, and health sciences John Wiley & Sons; 2013 5 20.

[43] de Winter AF, Visser L, Verhulst FC, Vollebergh WA, Reijneveld SA. Longitudinal patterns and predictors of multiple health risk behaviors among adolescents: the TRAILS study. Prev Med. 2016 3 31;84:76–82. doi: 10.1016/j.ypmed.2015.11.028 26656404 10.1016/j.ypmed.2015.11.028

[44] Jáuregui A, Villalpando S, Rangel-Baltazar E, Lara-Zamudio YA, Castillo-García MM. Physical activity and fat mass gain in Mexican school-age children: a cohort study. BMC pediatrics. 2012 7 28;12(1):109. 22839498 10.1186/1471-2431-12-109 PMC3441390

[45] Brault MC, Aimé A, Bégin C, Valois P, Craig W. Heterogeneity of sex-stratified BMI trajectories in children from 8 to 14years old. Physiol Behav. 2015 4 1;142:111–20. doi: 10.1016/j.physbeh.2015.02.001 25656690 10.1016/j.physbeh.2015.02.001

[46] Patte KA, Laxer R, Qian W, Leatherdale ST. Weight Perception and Weight-control Intention among Youth in the COMPASS Study. Am J Health Behav. 2016 9 1;40(5):614–23. doi: 10.5993/AJHB.40.5.8 27561864 10.5993/AJHB.40.5.8

[47] Hayward J, Millar L, Petersen S, Swinburn B, Lewis AJ. When ignorance is bliss: weight perception, body mass index and quality of life in adolescents. International journal of obesity. 2014 10 1;38(10):1328–34. doi: 10.1038/ijo.2014.78 24824556 10.1038/ijo.2014.78 PMC4189380

[48] van der Sluis ME, Lien N, Twisk JW, Steenhuis IH, Bere E, Klepp KI, Wind M. Longitudinal associations of energy balance-related behaviours and cross-sectional associations of clusters and body mass index in Norwegian adolescents. Public Health Nutr. 2010 10;13(10A):1716–21. doi: 10.1017/S1368980010002272 20883571 10.1017/S1368980010002272

[49] Leatherdale S, Rynard V. A cross-sectional examination of modifiable risk factors for chronic disease among a nationally representative sample of youth: are Canadian students graduating high school with a failing grade for health? BMC Public Health, 2012;13(1), 569. 10.1186/1471-2458-13-569 PMC3751757 23758659

[50] Nelson MC, Kocos R, Lytle LA, Perry CL. Understanding the perceived determinants of weight-related behaviors in late adolescence: a qualitative analysis among college youth. J Nutr Educ Behav. 2009 8 31;41(4):287–92. doi: 10.1016/j.jneb.2008.05.005 19508935 10.1016/j.jneb.2008.05.005

[51] Cairney J, Leatherdale ST, Faulkner GE. A longitudinal examination of the interrelationship of multiple health behaviors. Am J Prev Med. 2014 9 30;47(3):283–9. doi: 10.1016/j.amepre.2014.04.019 25145617 10.1016/j.amepre.2014.04.019

[52] Press, T. C. (2017, January 31). The Toronto Star. Retrieved March 10, 2017, from thestar.com: https://www.thestar.com/news/canada/2017/01/31/canada-pressing-forward-with-marijuana-legalization-amid-us-uncertainty.html

[53] Field AE, Gillman MW, Rosner B, Rockett HR, Colditz GA. Association between fruit and vegetable intake and change in body mass index among a large sample of children and adolescents in the United States. International journal of obesity. 2003 7 1;27(7):821–6. doi: 10.1038/sj.ijo.0802297 12821968 10.1038/sj.ijo.0802297

[54] Taylor WC, Sallis JF, Dowda M, Freedson PS, Eason K, Pate RR. Activity patterns and correlates among youth: differences by weight status. Pediatric Exercise Science. 2002 11;14(4):418–31.

[55] Roux AD. A glossary for multilevel analysis. J Epidemiol Community Health. 2002 8 1;56(8):588–94. doi: 10.1136/jech.56.8.588 12118049 10.1136/jech.56.8.588 PMC1732212

[56] Kuskowska-Wolk A, Karlsson P, Stolt M, Rössner S. The predictive validity of body mass index based on self-reported weight and height. Int J Obes. 1989;13(4):441–53. 2793299

[57] Flegal KM, Tabak CJ, Ogden CL. Overweight in children: definitions and interpretation. Health Educ Res. 2006 10 27;21(6):755–60. doi: 10.1093/her/cyl128 17071853 10.1093/her/cyl128

